# Exponential self-replication enabled through a fibre elongation/breakage mechanism

**DOI:** 10.1038/ncomms8427

**Published:** 2015-06-17

**Authors:** Mathieu Colomb-Delsuc, Elio Mattia, Jan W. Sadownik, Sijbren Otto

**Affiliations:** 1 Centre for Systems Chemistry, Stratingh Institute, University of Groningen, Nijenborgh 4, 9747 AG, Groningen, The Netherlands

## Abstract

Self-replicating molecules are likely to have played a central role in the origin of life. Most scenarios of Darwinian evolution at the molecular level require self-replicators capable of exponential growth, yet only very few exponential replicators have been reported to date and general design criteria for exponential replication are lacking. Here we show that a peptide-functionalized macrocyclic self-replicator exhibits exponential growth when subjected to mild agitation. The replicator self-assembles into elongated fibres of which the ends promote replication and fibre growth. Agitation results in breakage of the growing fibres, generating more fibre ends. Our data suggest a mechanism in which mechanical energy promotes the liberation of the replicator from the inactive self-assembled state, thereby overcoming self-inhibition that prevents the majority of self-replicating molecules developed to date from attaining exponential growth.

## Abstract (web summary)

Exponential replication is one of the requirements for molecular-level Darwinian evolution, but is difficult to achieve in synthetic systems. Here, the authors show a peptide-functionalized macrocycle that forms supramolecular fibres with exponential growth when subjected to mild agitation.

## Graphical abstract

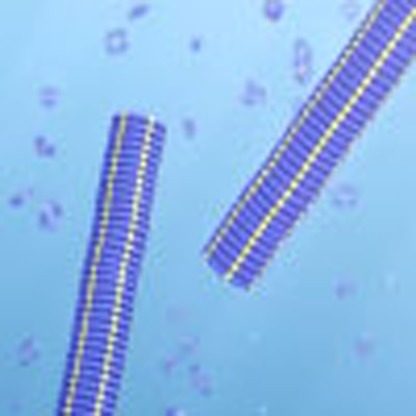

Replication of information-containing molecules is of key importance in life as we know it. Although in biology replication is mediated by complex biomolecular machinery, in the prebiotic world this process must have occurred through much simpler mechanisms. This postulate has spurred the development of relatively simple self-replicating and cross-replicating molecules^1^,^2^ based on nucleic acids^3^,^4^, peptides^5^,^6^ or fully synthetic structures^7^,^8^,^9^. The typical design of self-replicating systems is based on template-directed ligation of two halves of the replicator, to produce a noncovalent dimer of the autocatalyst. Subsequent dissociation of this duplex will liberate two replicators that can each mediate another round of replication, potentially enabling exponential growth of the replicator (Fig. 1a). However, such exponential replication is only rarely realized, because achieving sufficient duplex dissociation is in most cases problematic. Von Kiedrowski demonstrated that, when a significant proportion of the replicator resides in the inactive duplex state, replicator growth is typically parabolic; the reaction has an order of 0.5 in the autocatalyst^10^,^11^,^12^. Exponential growth occurs only when the order in autocatalyst is 1. This difference in replication kinetics has important consequences for evolutionary scenarios where several replicators compete for a common resource. Equations 1, 2 and describe the kinetics of a simple competition^11^,^12^,^13^:


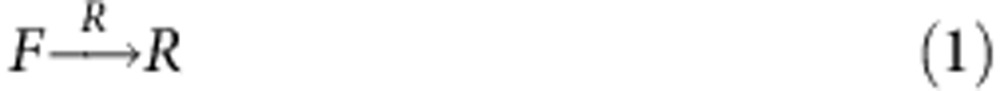



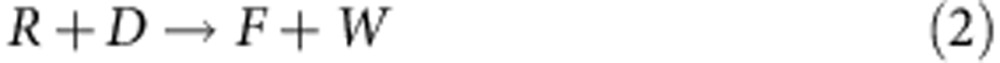



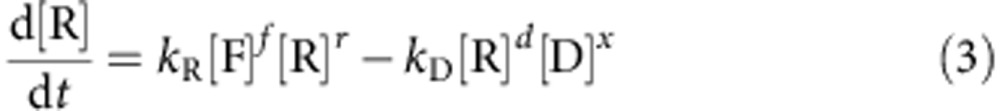


where *F* is a food molecule, *R* is a replicator, *D* is a destroying agent and *W* a waste molecule. The rate of replicator formation is given in by equation 3, in which *k*_R_ and *k*_D_ are the rate constants for the replication and destruction reaction, respectively. The order of the replication process in food and replicator are given by *f* and *r,* and the order of the destruction process in replicator and destroying agent are given by *d* and *x*, respectively. In order for competition to result in destruction of the weakest replicator (a necessary but not sufficient requirement for Darwinian evolution), the order in replicator in the replication process has to be higher than or equal to the order in replicator in its destruction reaction (that is, *r*≥*d*)^11^,^12^,^13^. In most plausible replicator destruction scenarios *d* equals 1, for example, by a bimolecular reaction with a destroying agent shown in equation 2 or by removal of products through flowing part of the solution out of the system. Therefore, for most common competition/destruction scenarios, an order in replicator of at least 1 (that is, exponential replication) is required to achieve Darwinian evolution. Although systems have been reported that move from parabolic towards exponential replication^14^,^15^,^16^,^17^, few systems have been reported which achieve *r*≥1 non-autonomously^18^,^19^,^20^ or autonomously^4^,^21^, but even in these examples no general mechanism for exponential growth was reported. The lack of design criteria for self-replicators capable of exponential growth constitutes a major problem that needs to be solved before approaches to Darwinian evolution of synthetic molecules can become mainstream.

We now report a new mechanism by which exponential replication can be achieved based on a fibre growth/breakage mechanism acting on self-assembling replicators. In principle, replication through this mechanism could give rise to exponential growth, as each time a fibre breaks this doubles the number of fibre ends. We now demonstrate experimentally that the replication process in our system is consistent with exponential growth. We then offer experimental insights into the mechanism of replication: a key role for fibre breakage is evident from the fact that the stirring rate influences both the rate of replication and the concentration of fibre ends. We demonstrate a direct correlation between average fibre length and replication rate. Furthermore, we show that fibre length distributions remain constant during replication, which is necessary in order to achieve exponential replication. Finally, we also confirm computationally that our hypothesized fibre elongation/breakage mechanism results in exponential replication and that the fibre breakage step is required for exponential growth.

## Results

### Replicator emergence from a dynamic combinatorial library

**Figure 1:**
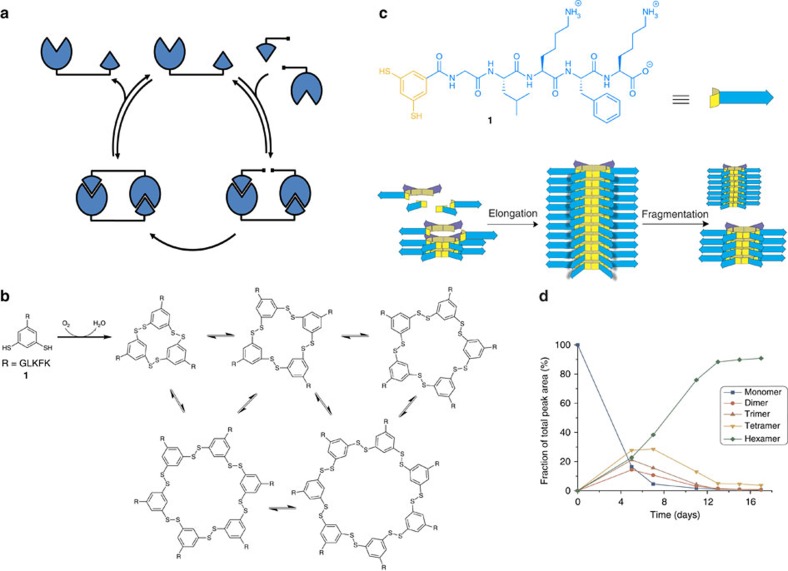
Replication mechanisms. (**a**) Traditional mechanism based on template-directed ligation of two replicator precursors. (**b**) Oxidation of building block **1** containing two thiol functionalities leads to a mixture of interconverting macrocycles. (**c**) Self-assembly of hexamers **1**_6_ results in the formation of fibres. Fibre fragmentation results in doubling of the number of fibre ends. (**d**) Species distribution as a function of time of a non-seeded dynamic combinatorial library made from 3.8 mM **1** in 50 mM borate buffer pH 8.1 stirred at 1,500 r.p.m.

This work builds on our previous observations on the emergence of replicators from dynamic combinatorial libraries^22^ arising from peptide building blocks, such as **1** (refs 23, 24). Oxidation of an aqueous solution of **1** gives rise to a continuously exchanging pool of disulfide macrocycles (Fig. 1b). Initially, cyclic trimers and tetramers dominate but, upon agitation, self-replicating hexamers emerge, which self-assemble into fibres and eventually become the major product in solution (Fig. 1d). Note that monomers, trimers or tetramers do not assemble into fibres. Hexamer growth is sigmoidal and seeding experiments confirmed that the growth process is autocatalytic. Shear stress is crucial for replication: in a non-agitated library trimers and tetramers are the only significant products, and no fibres could be observed by transmission electron microscopy (TEM) analysis of libraries dominated by those two species. The hexamer replicator only emerges when libraries are agitated. A mechanism for the replication process was proposed that involves two steps: fibre elongation by growth of the fibres from their extremities and breakage due to mechanically induced shear stress (Fig. 1c). Fibre growth by elongation may take place by sequestration of hexamer macrocycles from the macrocycle equilibrium, or through a mechanism in which fibre ends catalyse the formation of the hexamer macrocycle. Parallels exist between this mechanism and the nucleation-growth mechanisms of amyloid fibres, in which mechanically induced breakage can also play a role^25^,^26^,^27^,^28^,^29^. The hierarchical self-assembly process (monomers organizing into hexamers that then stack into fibres) is somewhat reminiscent of the fully non-covalent assembly of rosettes made from melamine derivatives and cyanuric acid, which have been proposed as prebiotic precursors to RNA analogues^30^,^31^. However, neither amyloids nor melamine rosettes involve covalent bond formation or self-replication.

### Experimental determination of the order in replicator

In the absence of replicator destruction, the rate law for replication (equation (3)) simplifies to:


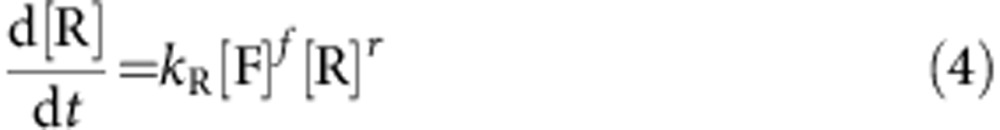


To determine the order in replicator *r*, equation 4 may be re-written as:


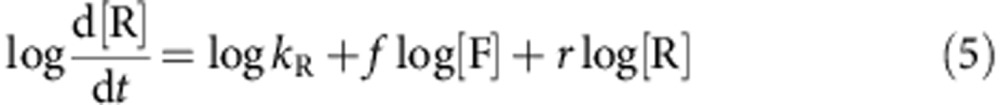


**Figure 2:**
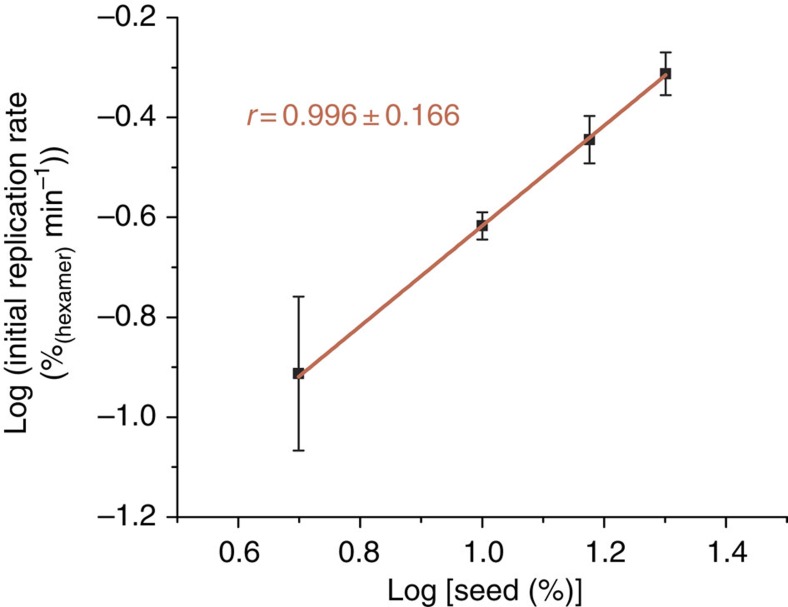
Experimental determination of the order in replicator. Initial replication rates against initial replicator concentration. Data points correspond to seed concentrations (expressed as concentration of **1**_6_) of 31, 63, 95 and 127 μM, respectively. The error bars on the data points correspond to one standard deviation for each seed concentration and the error on the slope is the standard deviation based on the complete set of measurements.

During the initial phase of growth, the concentration of food molecules *F* is approximately constant, hence log *k*_R_*+f* log[F] is constant. Following the methodology developed by von Kiedrowski, the replication order *r* may now be determined from the slope of a plot of the initial rate of replication (log d[R]/d*t*) versus log[R]^3^,^10^. We determined the initial rate of replication for different replicator concentrations through a set of seeding experiments. A series of identical samples rich in monomers, trimers and tetramers of **1** was prepared and seeded with different amounts of a stirred solution of pre-formed hexamer fibres. The initial rate of increase of the hexamer concentration was determined by ultra performance liquid chromatography (UPLC) analysis (under the chromatographic conditions, the fibres dissociate into their constituent hexamer macrocycles, which can be quantified from their ultraviolet–visible absorbance). The individual kinetic profiles are shown in Supplementary Fig. 1. The resulting plot of the initial rate of replication versus hexamer seed concentration is shown in Fig. 2, which allowed us to determine a value for the order in replicator *r* of 0.996±0.166. The error bars in Fig. 2 represent one standard deviation for each seed concentration (three to four measurements for each data point) and the error in *r* is a standard deviation based on the complete set of seeding experiments. Thus, within the experimental error, this system appears to be capable of exponential replication. However, given the experimental error on the value of *r*, we cannot exclude that replication is sub-exponential based on these experiments alone. To obtain additional evidence for exponential replication, we performed a detailed investigation to establish the replication mechanism. We then calculated the exact value of *r* associated with this replication mechanism through computer simulations (which are not subject to experimental errors).

### Experimental insights into the mechanism

To ascertain the validity of our postulated mechanism, we further investigated our system experimentally. We probed the role of fibre breakage in replication and the relationship between fibre end concentration and replicator concentration.

**Figure 3:**
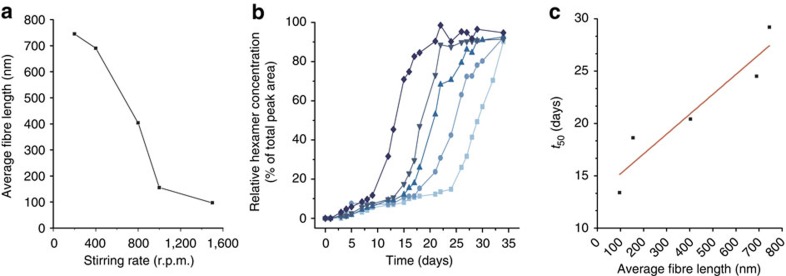
Influence of the stirring rate on fibre length and replication kinetics. (**a**) Average fibre length for different stirring rates. (**b**) Kinetics of replicator growth at various stirring rates (lighter to darker blue: 200, 400, 800, 1,000 and 1,500 r.p.m.). (**c**) Time needed for the replicator to represent 50% of the library material (*t*_50_), as a function of the average fibre length; the line represents a linear fit of the data.

First, the effects of stirring on the average fibre length and on the rate of replication were investigated. Fibre length distributions were determined using TEM for identical samples of pre-formed hexamer fibres that were subjected to different stirring rates. As expected, the average fibre length decreases substantially with increasing stirring rate, ranging from 745 nm at 200 r.p.m. to 97 nm at 1,500 r.p.m. (Fig. 3a; for details, see Supplementary Fig. 2 and Supplementary Table 1). Higher stirring rates also result in more efficient replication. Figure 3b shows the change in replicator concentration with time for different libraries stirred at rates ranging from 200 to 1,500 r.p.m. At higher stirring rates, the hexamers emerge faster than at lower rates of stirring.

The two previous results together imply that in systems with shorter fibres replication is more efficient; this is also evident from Fig. 3c where *t*_50_, that is, the time it takes for the hexamer to represent 50% of the total library material, is plotted against the average fibre length (values obtained from the data in Fig. 3b). Average fibre length and *t*_*50*_ do indeed appear to be correlated, as expected for a mechanism where fibre growth takes place from fibre ends; that is, when the same quantity of replicator is distributed over a larger number of shorter fibres (hence, more fibre ends), replication is more efficient than when it is distributed over a smaller number of longer fibres (hence, fewer fibre ends).

**Figure 4:**
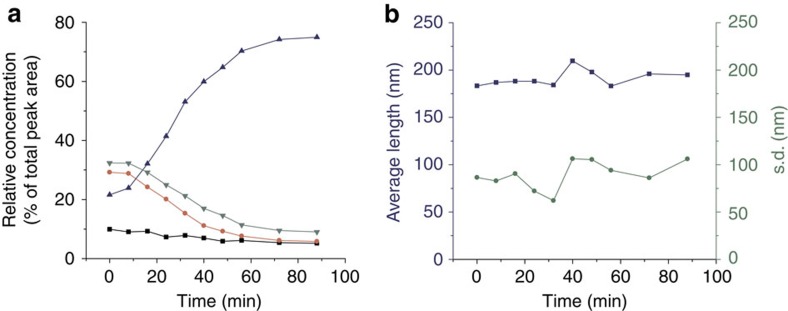
Change in replicator concentration and average fibre length during replication. (**a**) Change in product distribution with time of a library made from building block **1** (3.8 mM) stirred at 1,200 r.p.m. composed of **1** and cyclic trimer and tetramer seeded at *t*=0 min with 20% pre-formed hexamer fibres (monomer concentration as black squares, trimers as green triangles, tetramers as red circles, hexamers as blue triangles). (**b**) Average fibre length (blue squares) and associated standard deviation of the fibre length distribution (green circles) of this seeded library as a function of time (determined by TEM).

Finally, we also studied whether the average fibre length changed during the replication process. In fact, in a mechanism where replication takes place at the fibre ends, for replication to be exponential (that is, *r*=1), the average fibre length should not vary with time during replication. The concentration of fibre ends is only directly proportional to the total replicator concentration if the average fibre length is constant during replication. Note that, at a given amount of replicator, the concentration of fibre ends is independent of the fibre length distribution. This fact follows directly from the definition of the average fibre length, which equals the sum of the lengths of all fibres divided by the number of fibres. The first term is constant and determined by the replicator concentration (assuming all replicator to be incorporated into the fibres), whereas the second term equals half the number of fibre ends. We monitored product distribution by UPLC (Fig. 4a) and the average fibre length by TEM (Fig. 4b) over the course of the replication process in a partially oxidized library containing mainly monomer, trimer and tetramer, which was seeded at *t*=0 min with pre-formed hexamer fibres and stirred at 1,200 r.p.m.

Figure 4b shows that the average fibre length is essentially constant (see Supplementary Fig. 3 and Supplementary Table 2 for details), whereas the total fibre concentration increased approximately fourfold (21–75%). Thus, the system reaches a dynamic stationary state in fibre length in which fibre elongation is balanced by mechanically induced fibre breakage. Constant fibre length suggests a direct proportionality between replication rate and concentration of fibre ends, a prerequisite for exponential replication with *r*=1.

### Computational modelling of the elongation/breakage mechanism

**Figure 5:**
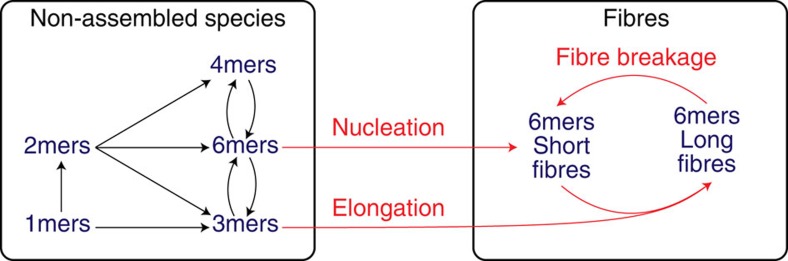
Computational model of the replicating system. Fibre elongation and fibre breakage could be toggled on or off in order to study their role in replication.

To corroborate that our proposed elongation/breakage mechanism gives rise to exponential growth of hexamer fibres, the replicating system was also studied computationally and the order in replicator was determined through numerical simulations that are unencumbered by experimental errors. The exchange reactions in solution and the fundamental steps for replication according to our hypothesis, that is, fibre nucleation, elongation and breakage, were included in a kinetic model (Fig. 5, see Supplementary Note 1 for more details), which was studied under a set of parameters. We used this model to address two key questions: first, does the elongation/breakage mechanism result in exponential replication, and if so, can we obtain a more accurate estimate of the order in replicator *r* than was possible experimentally? Second, is breakage necessary for exponential replication? Related to this second question we also set out to determine the value of *r* in a model that lacks breakage, but is otherwise identical to the original model.

The kinetic scheme, which has been employed for our numerical simulations, is depicted in Fig. 5 and the simulations are described in further detail in the Supplementary Note 2 (the full code is provided in Supplementary Method 1). It considers non-assembled species and self-assembled fibres of varying length, along with exchange processes and fibre elongation and breakage steps.

**Figure 6:**
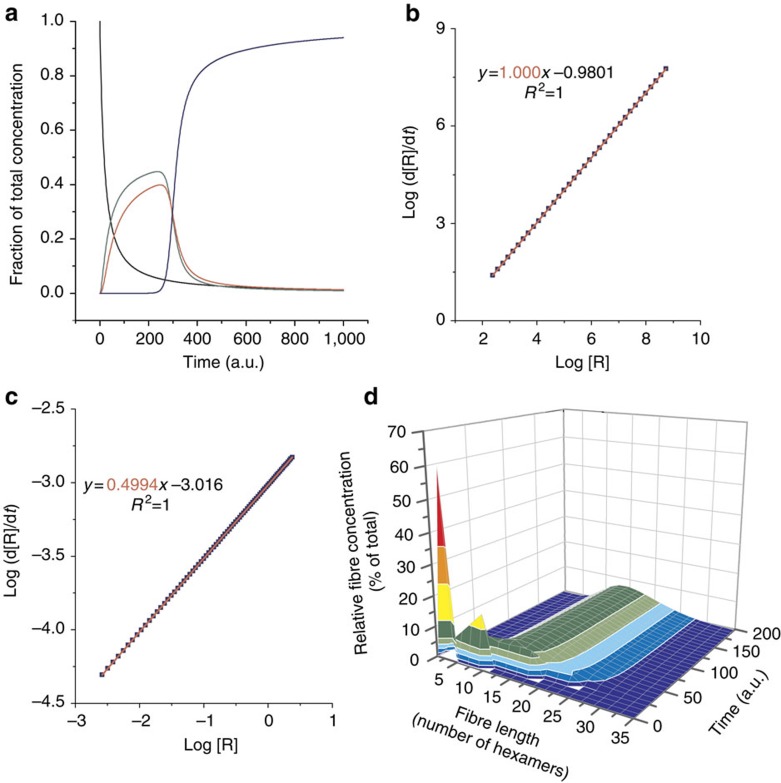
Computational studies on the growth/breakage mechanism. The numerical simulations show distinct kinetics in the presence of the breakage mechanism and in the absence thereof. (**a**) Typical kinetics observed in the numerical simulations of the growth/breakage mechanism (monomers in black, trimers in green, tetramers in red and hexamers in blue). (**b**) Computational determination of the order in replicator *r* in the case of a growth/breakage mechanism. (**c**) Computational determination of the replication order in the case of an elongation-only (breakage-free) mechanism. (**d**) Fibre length distribution as a function of time. After an initial transient regime, replication occurs at a steady-state length distribution.

As shown in Fig. 6a, simulations with the complete elongation/breakage mechanism were able to qualitatively reproduce the behaviour of the experimental system, where monomers are oxidized to trimers and tetramers via the formation of dimers (not shown for simplicity), before replication takes over and consumes the smaller macrocycles through the sigmoidal growth of hexamer species (Fig. 1d).

We determined the order in replicator *r* by using equation 5, which is only valid if the concentration of food molecules *F* is constant. In the present model, the supply of food molecules was kept constant by fixing the concentrations of trimers and non-assembled hexamers to a constant value throughout the simulation (equivalent to a system in which any consumed food molecules are instantly replenished from the surroundings). Figure 6b shows the central region of a plot of the logarithm of replication rate against the logarithm of replicator concentration (see Supplementary Note 2 and Supplementary Fig. 4 for the complete plot and further details). A linear least squares fit of the data yielded an order in replicator of *r*=1.000 confirming that a fibre elongation/breakage mechanism indeed results in exponential replication. Note that obtaining an observed order in replicator of 1 is only possible under conditions in which the production of replicator through the uncatalysed background reaction is negligible. For our family of self-replicators the background reaction is extremely slow: in the absence of agitation no significant amount of replicator **1**_6_ forms, even after 1 month.

To further ascertain the role of fibre breakage in the nature of the replication process, the system was also studied computationally under breakage-free conditions, that is, by deactivating the corresponding breakage pathway. Interestingly, the results, shown in Fig. 6c, display a replication order close to 0.5, that is, the value commonly associated to self-replicators that suffer from self-inhibition through duplex formation. Theoretical work by Stadler on replicators that form triplexes (while not considering mechanical breakage) concluded that also in these systems product inhibition leads to an order in replicator of 0.5 (ref. 32; see Supplementary Note 3 for further details). This resemblance in order in replicator between our system and the duplex or triplex systems is coincidental; it has a different origin than the ‘square root law' of autocatalysis typically associated with replicator dimerization. It may be rationalized as follows: in a mechanism in which new fibres nucleate continuously at a constant rate, but no fibres break, the rate of replication increases linearly with time as it depends on the number of fibre ends (d[R]/d*t*∼*t*). Furthermore, under these conditions it can be shown that the concentration of replicator increases proportionally to *t*^2^ ([R]∼*t*^2^) (see Supplementary Figs 5 and 6 for further details). Therefore, without fibre breakage, the rate of replication is proportional to the square root of the replicator concentration (d[R]/d*t*∼[R]^0.5^). Thus, the simulations reveal that in the absence of fibre breakage no exponential growth is obtained, highlighting the crucial role of fragmentation in exponential replication.

Finally, we also monitored the fibre length distributions over time during the simulations. As shown in Fig. 6d, after a short initial transient phase, the length distribution remained constant throughout the replication process, similar to the behaviour we observed experimentally (see Supplementary Fig. 7).

## Discussion

We have demonstrated that replicators which, upon replication, assemble into large but fragile aggregates may be liberated by fracturing the aggregate through mild mechanical forces. Such fragmentation can overcome replicator self-inhibition and, most importantly, enables exponential replication, consistent with our experimental data and evident from numerical simulations. Notably, our simulations also indicate that, without breakage, the system reverts back to obeying the ‘square root law of autocatalysis', albeit through a mechanism different from that postulated for duplex-forming replicators.

The growth/breakage mechanism may well provide a general solution to the auto-inhibition problem almost inherently associated with self-replication. We speculate that the replicators developed by Ashkenasy^21^ might well work by a similar mechanism. Our mechanistic understanding gives clear guidance that may be employed to successfully design new exponential replicators, which opens up realistic prospects of achieving Darwinian evolution in a purely synthetic chemical system. The next step in this direction involves developing conditions in which replication and destruction operate in parallel, giving rise to a dynamic kinetic stability regime^33^ that is characteristic for life. Work in this direction is currently taking place in our laboratory.

## Methods

### Library preparation and monitoring

Dynamic combinatorial libraries were prepared by dissolving building block **1**, obtained from Cambridge Peptides, in a 50 mM pH 8.1 potassium borate buffer to a final concentration of 3.8 mM. The pH of the resulting solution was adjusted to 8.1–8.2 by addition of small amounts of a 2.0 M KOH solution. All libraries were contained in HPLC vials (12 × 32 mm) tightly closed with Teflon-lined snap caps. The libraries were stirred using a Teflon-coated magnetic stirrer bar (5 × 2 mm, obtained from VWR), on an IKA RCT basic stirrer hotplate at 1,200 r.p.m. unless otherwise specified. Library compositions were monitored by quenching 2.0 μl samples of the library in 98 μl of a solution of doubly distilled H_2_O containing 0.6% trifluoroacetic acid, in a glass UPLC vial and injecting 5.0 μl of this sample on the UPLC. For samples that were monitored over time, it was confirmed that the total peak area in the UPLC chromatograms remained constant.

### UPLC-MS analysis

UPLC analyses were performed on a Waters Acquity UPLC I-class system equipped with a photodiode array detector. All analyses were performed using a reversed-phase UPLC column (Aeris Widepore 3.6 μm XB-C18 150 × 2.10 mm, purchased from Phenomenex). Ultraviolet absorbance was monitored at 254 nm. Column temperature was kept at 35 °C. UPLC-MS was performed using a Waters Acquity UPLC H-class system coupled to a Waters Xevo-G2 TOF. The mass spectrometer (MS) was operated in the positive electrospray ionization mode. Injection volume was 5 μl of a 3.8 mM library subjected to a 1:50 dilution in a solution of 0.6 v% of trifluoroacetic acid in doubly distilled water. Eluent flow was 0.3 ml min^−1^; eluent A: UPLC grade water (0.1 v% trifluoroacetic acid); eluent B: UPLC grade acetonitrile (0.1 v% trifluoroacetic acid). For full method, see Supplementary Table 3. For UPLC-MS compound identification see Supplementary Table 4.

### Seeding experiments

A library was prepared by dissolving 3.8 mM **1** in 50 mM borate buffer at pH 8.2. The library was then oxidized up to 70% using a freshly prepared solution of sodium perborate (38 mM, pH 8.0). The composition of the mixture was at this point: 25% monomer **1**, 5% linear dimer (**1**)_2_, 35% cyclic trimer (**1**)_3_ and 35% cyclic tetramer (**1**)_4_. The resulting solution was split into four samples of 200 μl and each one was then seeded with a pre-formed library rich in the hexamer of **1** (which had been continuously stirred at 1,200 r.p.m.), by adding, respectively, 5.0, 10, 15 and 20 mol% of hexamer (the mol% was calculated as equivalents of **1** in the hexamer relative to equivalents of **1** in the library). The library was then stirred at 1,200 r.p.m. and the change in hexamer concentration was monitored by sampling every 8 min as described above.

### Experiments at different stirring rates

A library was prepared by dissolving 3.8 mM **1** in 50 mM borate buffer at pH 8.2. It was then split into five samples of 200 μl, that were each placed in an HPLC vial (12 × 32 mm), which was tightly closed with a Teflon-lined snap cap and which contained a Teflon-coated magnetic stir bar 5 × 2 mm. The libraries were continuously stirred, each of them at identical positions on different stirring plates, all of the same brand (IKA RCT basic stirrer hotplate), at rates of 200, 400, 800, 1,000 and 1,500 r.p.m. The libraries were regularly sampled for UPLC monitoring.

### Negative staining TEM

An aliquot of a sample taken from a peptide library was diluted 40 times in doubly distilled water. Shortly thereafter, a small drop of the diluted sample was deposited on a 400 mesh copper grid covered with a thin carbon film. After 30 s, the droplet was blotted on filter paper. The sample was then stained with a solution of uranyl acetate deposited on the grid and blotted on filter paper after 30 s. The grids were observed in a Philips CM120 electron microscope operating at 120 kV. Images were recorded on a slow scan CCD camera.

### Fibre length measurements

TEM micrographs were analysed using ImageJ. A scale was put on each micrograph according to its magnification, and an average length of each sample was determined by measuring fibres from the micrograph, using the measuring tool of ImageJ. The data were then transferred and analysed using MS Excel.

## 

## Additional information

**How to cite this article**: Colomb-Delsuc, M. *et al.* Exponential self-replication enabled through a fibre elongation/breakage mechanism. *Nat. Commun.* 6:7427 doi: 10.1038/ncomms8427 (2015).

## Supplementary Material

Supplementary Information Supplementary Figures 1-7, Supplementary Tables 1-4, Supplementary Notes 1-3 and Supplementary Methods
