# Supplementary material for: Exponential self-replication enabled through a fibre elongation/breakage mechanism
Source: Nat Commun. 2015 Jun 17;6:7427. doi: 10.1038/ncomms8427 (PMC4557357; doi:10.1038/ncomms8427)
Supplement: Supplementary Information — Supplementary Figures 1-7, Supplementary Tables 1-4, Supplementary Notes 1-3 and Supplementary Methods [file ncomms8427-s1.pdf]

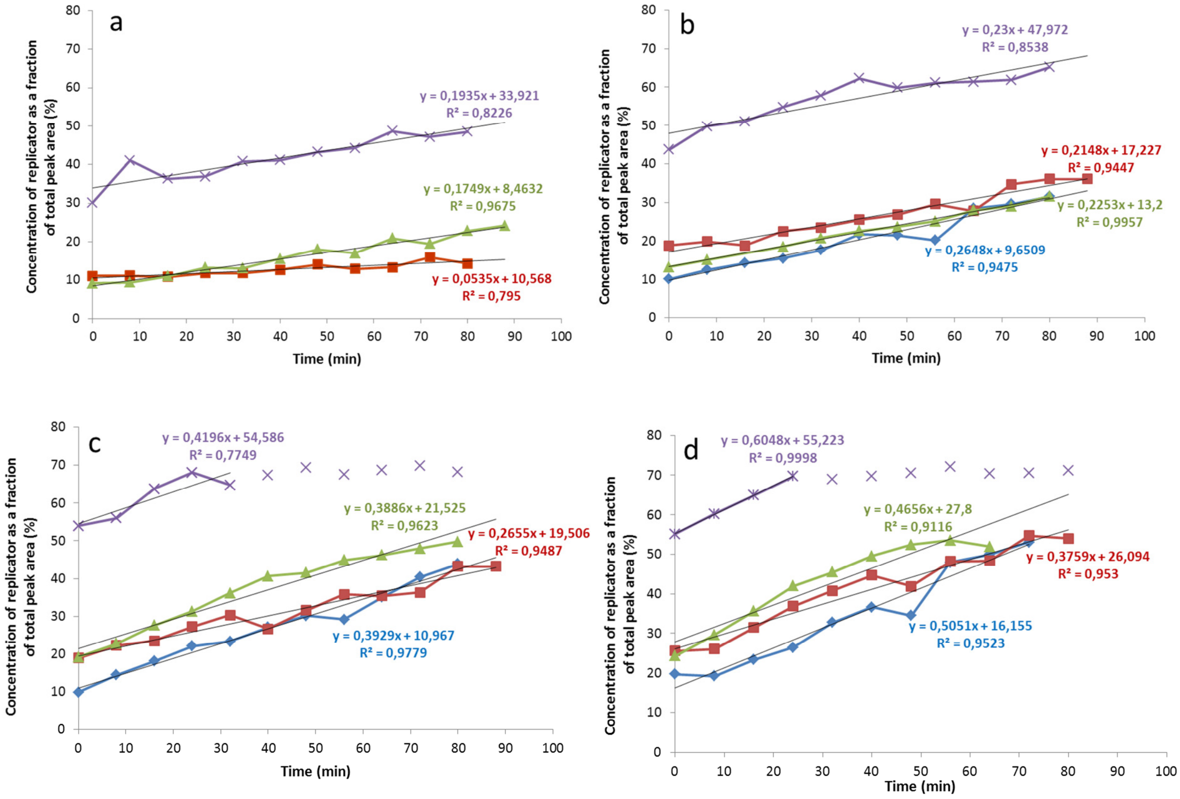


**Supplementary Figure 1 | Kinetics of replication in seeded libraries.** Libraries were seeded with different amounts of seed, specifically 5 mol% (a), 10 mol% (b), 15 mol% (c) and 20 mol% (d). Each color corresponds to a set of seeding experiments done on the same batch of library. Different colors represent replicas of the same experiment done on different batches of library. One set of measurements (shown in purple) gave an apparent initial replication concentration higher than for the other sets of experiments, leading to a faster saturation when seeded at 15 mol% and 20 mol%. For this reason, the initial replication rate was determined using the data points before a plateau was reached.

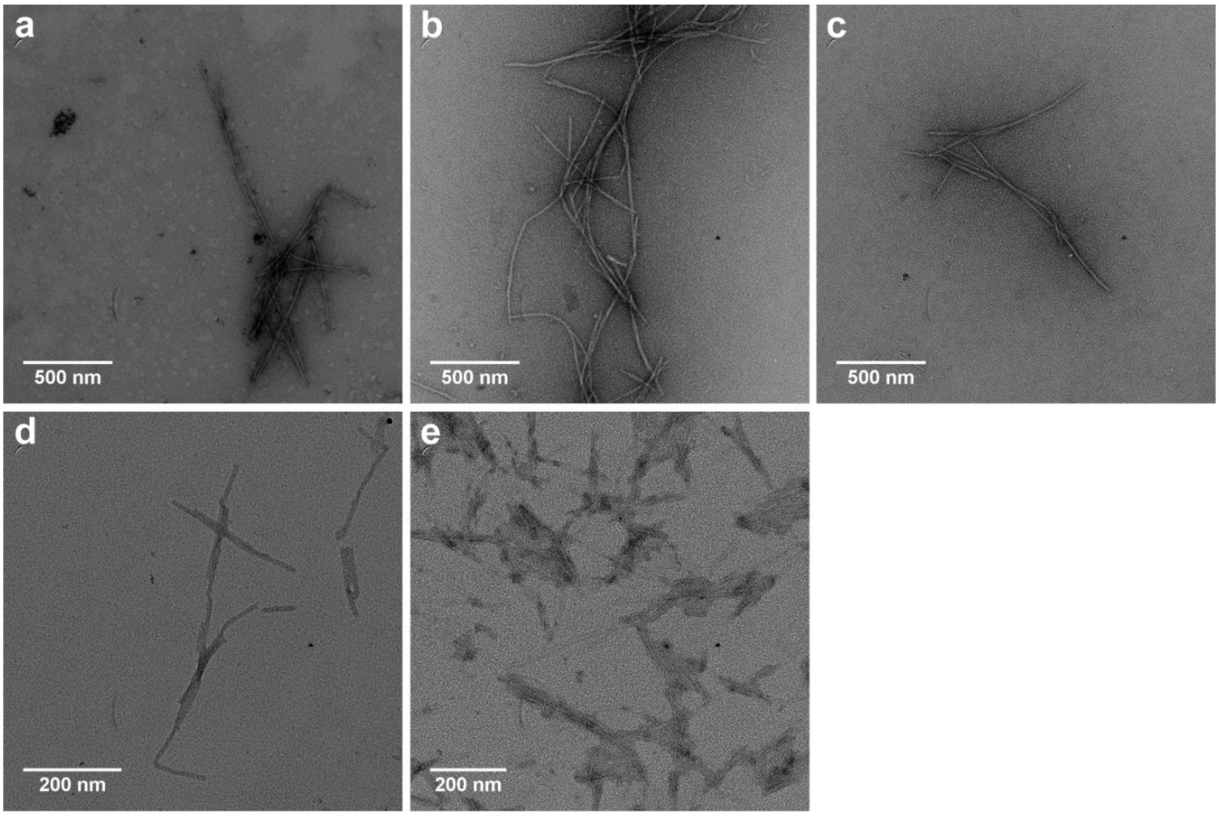


**Supplementary Figure 2 | TEM analysis of fibre length distributions at different stirring rates.** A set of libraries made from 3.8 mM solutions of building block **1** was stirred at different rates. After complete oxidation, and once the hexamer prevailed in all libraries, TEM measurements were made. The micrographs correspond to stirring rates of 200 rpm (a), 400 rpm (b), 800 rpm (c), 1000 rpm (d) and 1500 rpm (e).

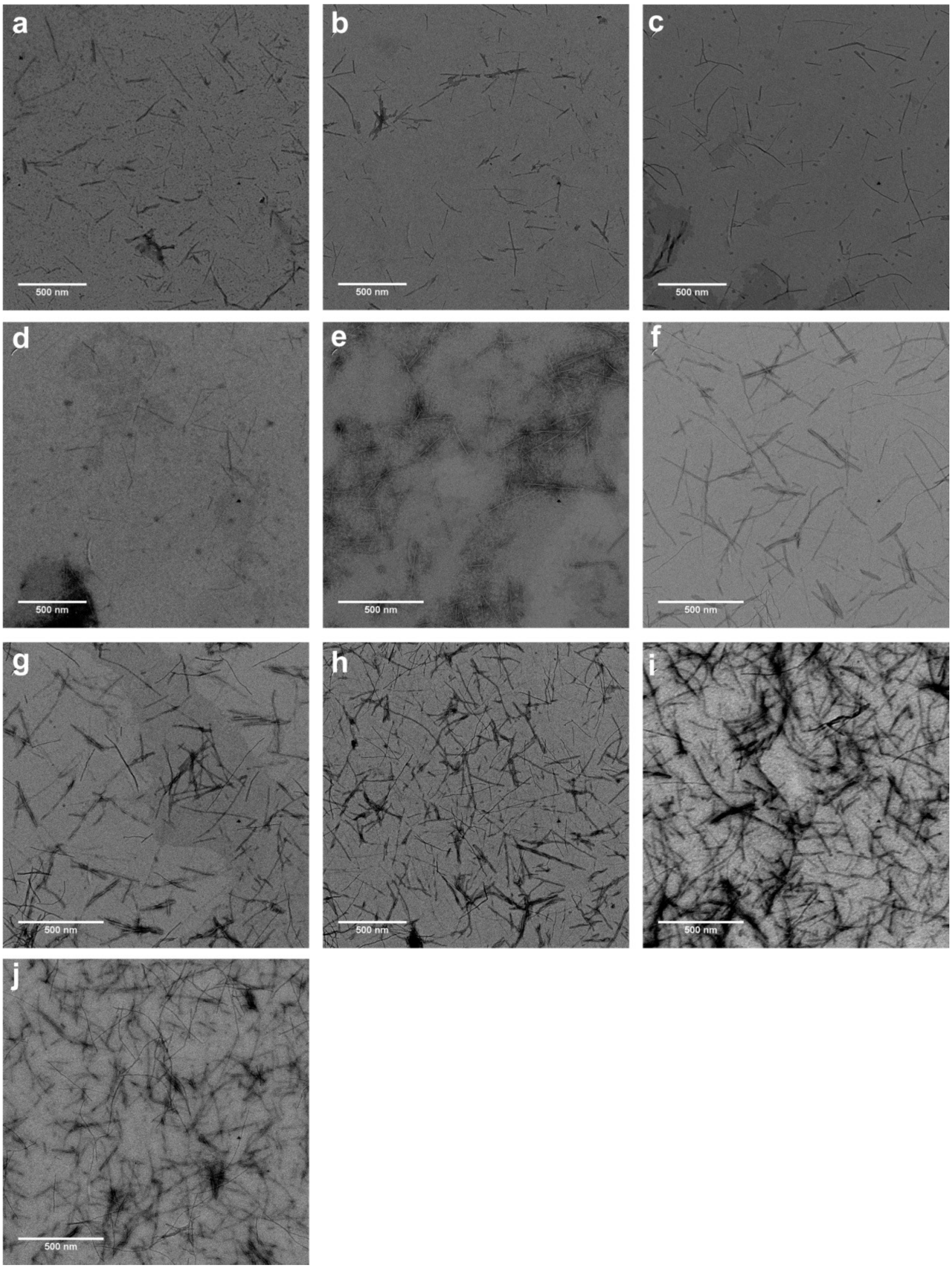


**Supplementary Figure 3 | TEM analysis of library samples during replication.** A library made from building block **1** (3.8 mM) was seeded with 20 mol % of a sample rich in hexamer of **1** and observed at different times after the addition of the seed. The micrographs correspond to times of 0 min (a), 8 min (b), 16 min (c), 24 min (d), 32 min (e), 40 min (f), 48 min (g), 56 min (h), 72 min (i) and 88 min (j). The sample was stirred at 1200 rpm for the entire duration of the experiment.

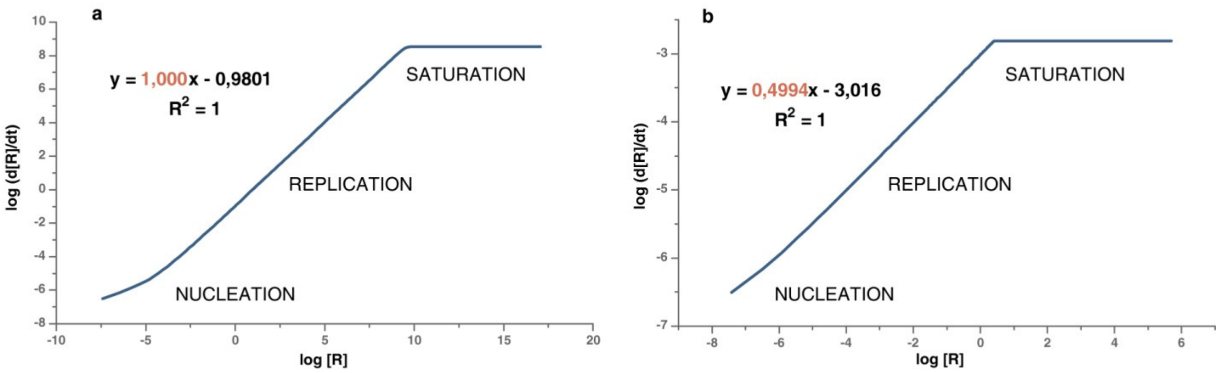


**Supplementary Figure 4 | Computational results: the growth/breakage mechanism vs. removal of breakage**. Three regions named "nucleation", "replication" and "saturation" are identified. During nucleation, the zero-order nucleation process is significant relative to the replication process. Replication involves instead autocatalytic growth of hexamers; this is the region used for data fitting in order to determine the replication order. Saturation is due to different reasons in the two illustrated cases. **a**, In the case of a growth/breakage mechanism, saturation is due to the constant building block supply being no longer sufficient to sustain exponentially faster replication. **b**, In the case of an elongation-only (breakage-free) mechanism saturation is due to reversal to linear growth due to reaching maximum fibre length (see Figs S6 and S7). This does not happen with active breakage, as the latter ensures continuous shortening of longer fibres.

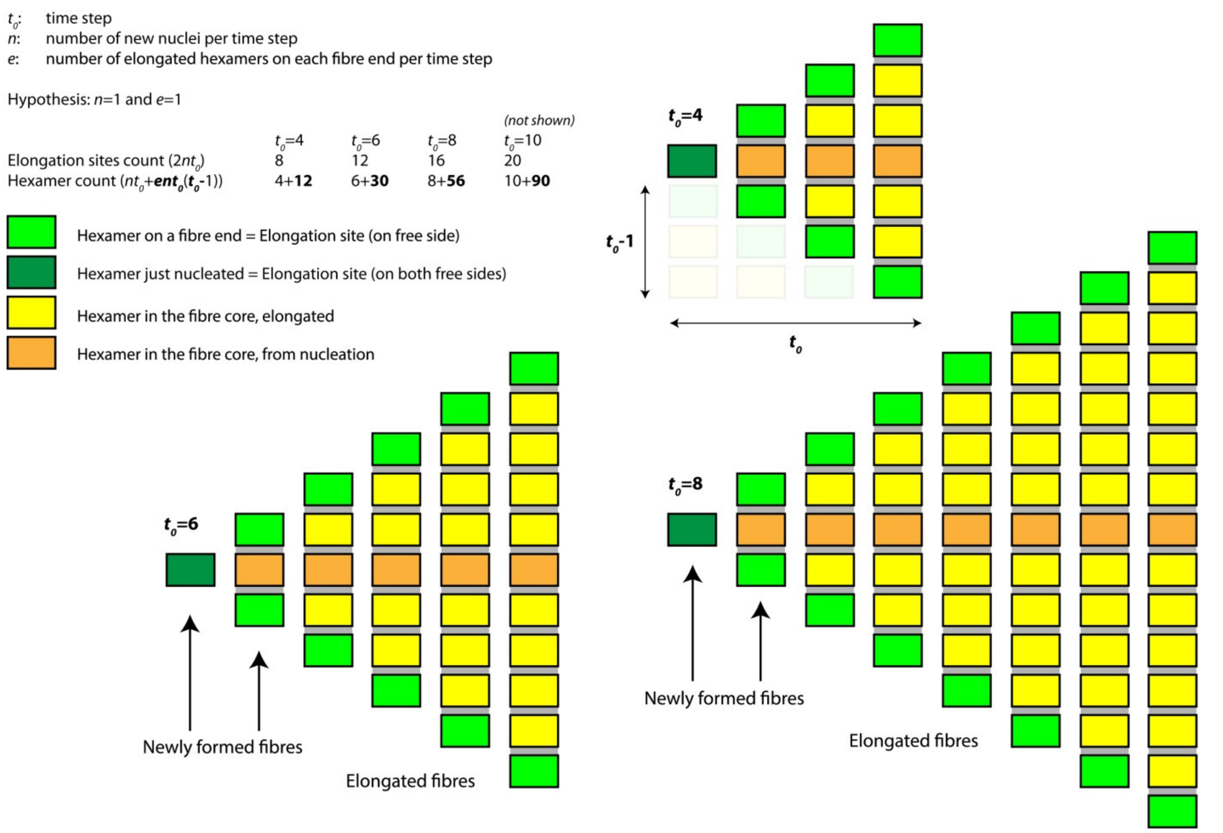


**Supplementary Figure 5 | Evolution of the fibre population without the fibre breakage mechanism.** Evolution of the fibre population at different time steps in numerical simulations which do not include the breakage mechanism (e=1, n=1, constant trimer supply). The number of elongation sites responsible for replication increases linearly with time, whereas the total hexamer count increases with the square of time, thereby making replication rate depend upon the square root of the total replicator concentration (r=0.5), at sufficiently high replicator concentrations.

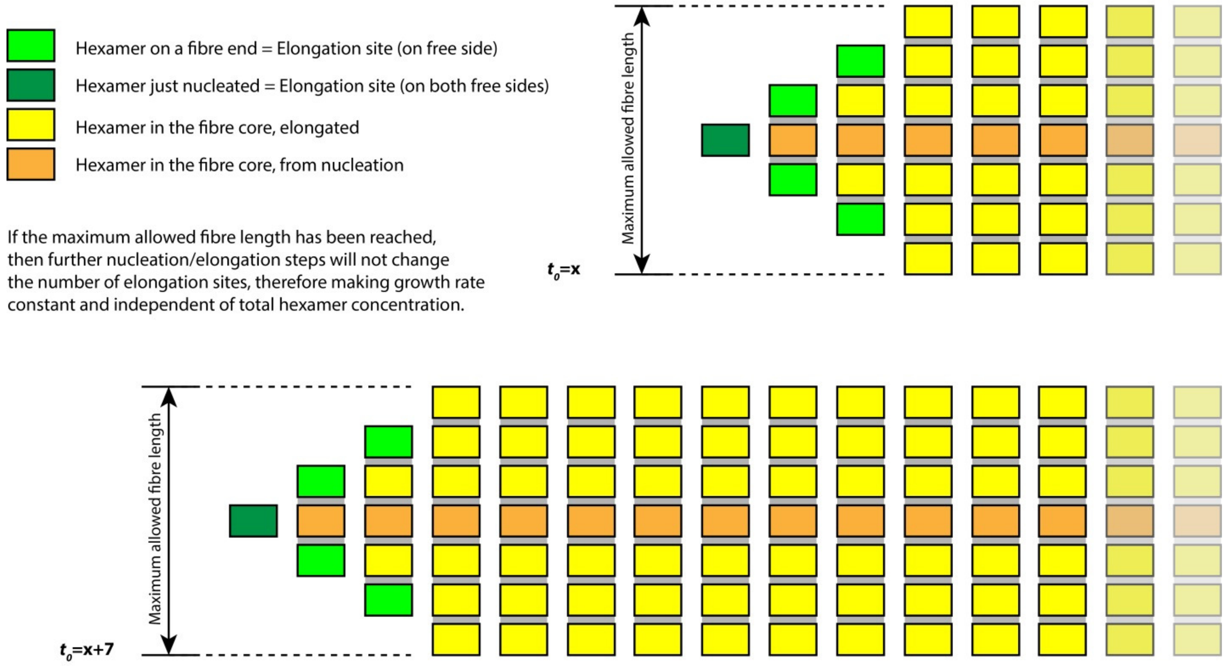


**Supplementary Figure 6 | Evolution of the fibre population without the fibre breakage mechanism, after maximum allowed fibre length has been reached.** Evolution of the fibre population at two different time steps in numerical simulations which do not include fibre breakage (e=1, n=1, constant trimer supply), after the maximum allowed fibre length has been reached. The growth rate is now independent of total hexamer concentration.

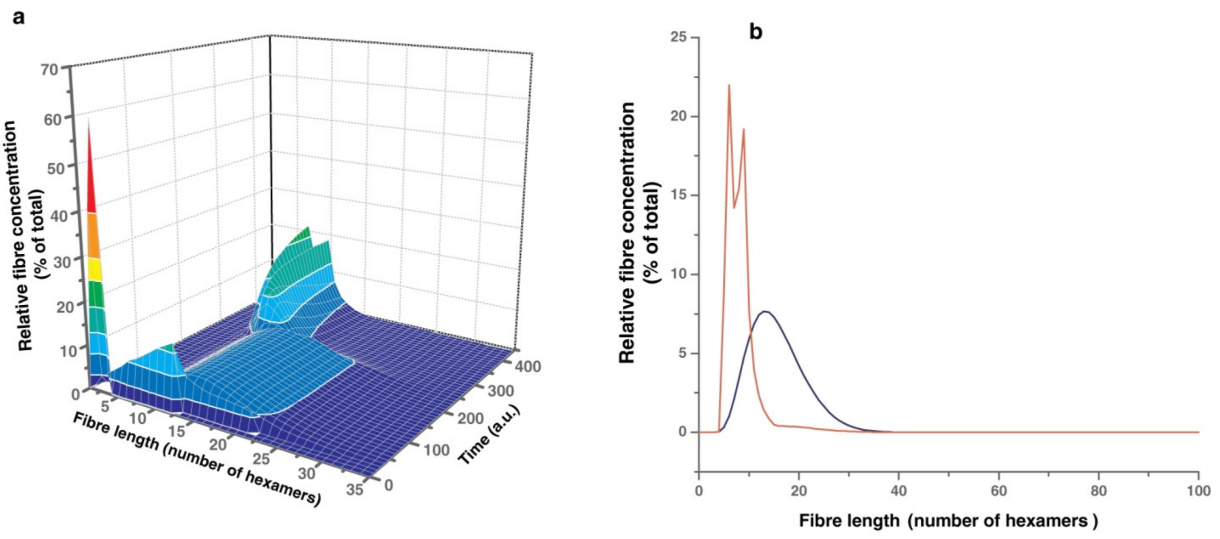


**Supplementary Figure 7 | Fibre length distribution in the replication and saturation phases, with breakage.** During replication the fibre length distribution remains constant , but changes in the saturation regime. **a**, Fibre length plot including the three different regions of the replication process. After a short transient phase the average fibre length remains constant during the exponential replication phase, but decreases steadily with time in the saturation regime. **b**, 2D plot of the fibre length distribution during (blue) and after (red) the exponential replication process. During the saturation phase a distribution with a lower average fibre length develops compared to the replication phase, due to fibre growth being relatively slower than fibre breakage in the saturation regime with respect to the exponential replication regime. Furthermore, in the saturation regime, the fibre length distribution steadily varies over time, unlike what happens in the replication regime, where the constant fibre length distribution ensures exponential growth of the fibre population.

| stirring rate (rpm) | average length (nm) | statistical sample size (number of measurements) |
|---|---|---|
| 200 | 745 | 37 |
| 400 | 690 | 35 |
| 800 | 404 | 38 |
| 1000 | 155 | 77 |
| 1500 | 97 | 30 |

**Supplementary Table 1 | Average fibre length at different stirring rates.** The number of length measurements for each sample at different stirring rates is also shown.

| time (min) | average length (nm) | statistical sample size (number of measurements) |
|---|---|---|
| 0 | 183,4 | 81 |
| 8 | 186,8 | 88 |
| 16 | 188,2 | 106 |
| 24 | 188,2 | 84 |
| 32 | 184,1 | 117 |
| 40 | 209,6 | 118 |
| 48 | 197,7 | 95 |
| 56 | 183,0 | 126 |
| 72 | 196,0 | 33 |
| 88 | 194,9 | 68 |

**Supplementary Table 2 | Variation of the average fibre length during replication.** The number of length measurements for each sample at different times during replication after the library was seeded with 20 mol % of a sample rich in hexamer of **1**.

| time (min) | %A | %B |
|---|---|---|
| 0,0 | 90,0 | 10,0 |
| 1,0 | 90,0 | 10,0 |
| 1,3 | 75,0 | 25,0 |
| 3,0 | 72,0 | 28,0 |
| 11,0 | 69,0 | 31,0 |
| 11,5 | 5,0 | 95,0 |
| 12,0 | 5,0 | 95,0 |
| 12,5 | 90,0 | 10,0 |
| 15,0 | 90,0 | 10,0 |

**Supplementary Table 3 | UPLC method.** Eluent gradient used for UPLC analysis of libraries formed from building block **1** where A: UPLC grade water (0.1 v% trifluoroacetic acid); eluent B: UPLC grade acetonitrile (0.1 v% trifluoroacetic acid).

| Compound no. | Retention time / min | m/z calculated | m/z observed |
|---|---|---|---|
| **1** | 6.6 | 760.35 $[M+H]^{1+}$, 380.68 $[M+2H]^{2+}$ | 760.32 $[M+H]^{1+}$, 380.65 $[M+2H]^{2+}$ |
| (**1**)$_2$ | 7.2 | 1517.7 $[M+H]^{+}$, 759.4 $[M+2H]^{2+}$, 506.6 $[M+3H]^{3+}$, 380.2 $[M+5H]^{3+}$ | 1517.5 $[M+H]^{+}$, 759.6 $[M+2H]^{2+}$, 506.8 $[M+3H]^{3+}$, 380.5 $[M+5H]^{3+}$ |
| (**1**)$_3$ | 9.9 | 1137.50 $[(M+1)+2H]^{2+}$, 759.01 $[(M+2)+3H]^{3+}$, 569.25 $[(M+1)+4H]^{4+}$, 455.61 $[(M+1)+5H]^{5+}$ | 1137.48 $[(M+1)+2H]^{2+}$, 758.98 $[(M+2)+3H]^{3+}$, 569.23 $[(M+1)+4H]^{4+}$, 455.58 $[(M+1)+5H]^{5+}$ |
| (**1**)$_4$ | 8.6 | 1516.67 $[(M+2)+2H]^{2+}$, 1011.45 $[(M+2)+3H]^{3+}$, 758.59 $[(M+1)+4H]^{4+}$ | 1516.64 $[(M+2)+2H]^{2+}$, 1011.42 $[(M+2)+3H]^{3+}$, 758.56 $[(M+1)+4H]^{4+}$ |
| (**1**)$_6$ | 9.1 | 1516.67 $[(M+3)+3H]^{3+}$, 1137.75 $[(M+3)+4H]^{4+}$, 910.80 $[(M+5)+5H]^{5+}$, 759.00 $[(M+4)+6H]^{6+}$ | 1516.62 $[(M+3)+3H]^{3+}$, 1137.72 $[(M+3)+4H]^{4+}$, 910.77 $[(M+5)+5H]^{5+}$, 758.99 $[(M+4)+6H]^{6+}$ |

**Supplementary Table 4 | UPLC-MS compound identification.**

**Supplementary Note 1 | Computational model**

The reaction network depicted in Figure 5 of the main text considers the main species detected experimentally in the library solutions, including monomers, linear dimers, cyclic trimers, tetramers and, notably, hexamers, together with fibres made from stacks of hexamers, up to an arbitrary maximum length (of 10 or 100 hexamers stacked in a single fibre). Concentrations, in arbitrary units, are monitored against time for each of these species, including fibres for each possible length up to the maximum allowed length. Processes in the system fall in two categories. First, oxidation reactions, such as linear dimer formation from monomers, cyclic trimer formation from monomers and linear dimers, cyclic tetramer formation from linear dimers and cyclic hexamer formation from linear dimers and cyclic tetramers. Second, fast equilibration processes among the oxidized species, i.e. between cyclic  trimers, tetramers and hexamers. The wiring of the reaction network, in absence of experimental evidence, is partly arbitrary, i.e. it is not possible to be certain that, e.g. tetramers are formed by direct oxidation of two dimers rather than oxidation of a monomer and a trimer; however, equilibria in solution ensure that different underlying pathways result in the same kinetic outcome when focusing on replication behaviour. Fibres nucleate from individual hexamers; they can be elongated from trimers, transforming a fibre of length $n$ (number of stacked hexamers) into a fibre of length $n+1$. The choice of growth from trimers is also arbitrary; however, trimers and tetramers also equilibrate fast. Finally, a fibre breakage process ensures that longer fibres are converted into smaller fibres, in the process active due to shear stress in our experimental systems. Only breakage of fibres in half (or breakage of $(2n+1)$-long fibres into $n$-fibres and $n+1$-fibres) was considered. Short fibres up to a threshold length (5 hexamers for simulations with maximum length of 10 hexamers and 10 hexamers for simulations with maximum length of 100 hexamers) were not allowed to break. Beyond the threshold length the breakage rate was set to increase linearly with fibre length, from zero probability of breakage at the next time step at the threshold length up to total certainty of breakage at the next time step for fibres of maximum length.

**Supplementary Note 2 | ODE and implementation of the model**

The computational model was developed and accordingly implemented in C++. A solver for the set of ODEs (Ordinary Differential Equations) modelling the replicating system was coded explicitly at the programming language level in order to properly account for general mass balance and positive concentration constraints. At any point of the simulation, a "configuration" of the system is kept track of and a new configuration for the current time step is computed by calculating incremental variations through numerical integration of the set of ODEs. The new configuration subsequently becomes the current one for the next time step. Other parameters are kept track of for more than one time step in order to ensure better approximation of the calculations. For example, the total hexamer concentration for three time steps is needed in order to calculate an $O(t^2)$ approximation (where $t$ is the duration of the time step) of the replication rate for the current time step. A configuration typically includes concentrations of monomers, linear dimers, cyclic trimers, tetramers, non-assembled hexamers and the concentration of fibres for every fibre length from a nucleus of 2 hexamers in a fibre up to a maximum length (i.e., number of hexamers in the same fibre).

The code is tunable on a number of parameters, among which the following:

- Number of time steps that the simulation is run for
- Maximum possible length (in number of hexamers) of individual fibres
- Monomer initial concentration
- Trimer initial concentration
- Non-assembled hexamer initial concentration
- Flag to keep the monomer concentration steady (constant along the simulation)
- Flag to keep the trimer concentration steady
- Flag to keep the non-assembled hexamer concentration steady
- Rate constants for the processes among the non-assembled species (described below)
- Rate constant for nucleation of hexamer fibres from non-assembled species
- Breakage probability profile against fibre length
- Rate constant for sequestration of non-assembled hexamers on fibre ends
- Rate constant for elongation of fibres from smaller macrocycles
- Flag to activate/deactivate fibre nucleation (if not active, artificial initial seeding is implemented)
- Flag to activate/deactivate fibre breakage

The prototypes of the functions implemented in the software are as follows:

```
void simulate(float *,configuration *);
void cfgcopy(configuration *,configuration *);
void generatebp(float *);
configuration *create();
void destroy(configuration *);
void createpath(char *);
void write(long,FILE *,configuration *,float *,float *,float *);
void parse(FILE *,char *);
```

While the **main()** cycles the time steps of the simulation and **simulate**(float *,configuration *) integrates the system of ODEs, the other functions serve different other fundamental roles, e.g., allocation/deallocation of memory for the simulation, and outputting/parsing of the results.

The system of ODEs is integrated within the **simulate** function. Much attention is dedicated to implementing a solver for this set of equations which respects mass balance and positive concentration constraints. The system includes the kinetic equations described for each individual process in the following (numbers are used to indicate the corresponding oligomers, i.e., 1 for monomers, 2 for dimers, …, $6_{na}$ for non-assembled hexamers, $6_i$ for hexamers within fibres of length *i*, etc.):

$$-\frac{d_{12}[1]}{dt} = 2\frac{d_{12}[2]}{dt} = k_{12}[1]^2$$

$$-\frac{d_{23}[2]}{dt} = -\frac{d_{23}[1]}{dt} = \frac{d_{23}[3]}{dt} = k_{23}[1][2]$$

$$-\frac{d_{24}[2]}{dt} = 2\frac{d_{24}[4]}{dt} = k_{24}[2]^2$$

$$-\frac{d_{36}[3]}{dt} = 2\frac{d_{36}[6_{na}]}{dt} = k_{36}[3]^2$$

$$-\frac{d_{46}[4]}{dt} = -\frac{d_{46}[2]}{dt} = \frac{d_{46}[6_{na}]}{dt} = k_{46}[2][4]$$

$$-\frac{d_{63}[6_{na}]}{dt} = \frac{1}{2}\frac{d_{63}[3]}{dt} = k_{63}[6_{na}]$$

$$-\frac{d_{64}[6_{na}]}{dt} = \frac{2}{3}\frac{d_{64}[4]}{dt} = k_{64}[6_{na}]$$

$$-\frac{1}{i-1}\frac{d_{sequestration}[6_{i-1}]}{dt} = -\frac{d_{sequestration}[6_{na}]}{dt} = \frac{1}{i}\frac{d_{sequestration}[6_i]}{dt} = k_{sequestration}[6_{i-1}]\frac{2}{i-1}[6_{na}]$$

Where $[6_{i-1}]\frac{2}{i-1}$ is the concentration of fibre ends for fibres of length *i-1* calculated from the corresponding concentration of hexamers which form fibres of such length.

$$-\frac{2}{i-1}\frac{d_{elongation}[6_{i-1}]}{dt} = -\frac{d_{elongation}[3]}{dt} = \frac{2}{i}\frac{d_{elongation}[6_i]}{dt} = k_{elongation}[6_{i-1}]\frac{2}{i-1}[3]^2$$

$$-\frac{d_{nucleation}[6_{na}]}{dt} = \frac{d_{nucleation}[6_2]}{dt} = k_{nucleation}[6_{na}]^2$$

$$-\frac{d_{breakage}[6_i]}{dt} = \frac{d_{breakage}\left[6_{\frac{i}{2}}\right]}{dt} = k_{breakage(i)}[6_i]$$

$$-\frac{d_{breakage}[6_i]}{dt} = \frac{2i}{i-1}\frac{d_{breakage}\left[6_{\frac{i-1}{2}}\right]}{dt} = \frac{2i}{i+1}\frac{d_{breakage}\left[6_{\frac{i-1}{2}+1}\right]}{dt} = k_{breakage(i)}[6_i]$$

The latter two equations describe the breakage rates for fibres of even and odd length, respectively.

Hence, the full differential model for our system is the following (all possible fibre lengths *i* higher than 2 should be considered), where the technical detail about fibres of odd length *i* being broken into fibres of length *(i-1)/2* and *(i-1)/2+1* has been neglected for clarity (but is incorporated into the simulations):

$$-\frac{d[1]}{dt} = k_{12}[1]^2 + k_{23}[1][2]$$

$$-\frac{d[2]}{dt} = -\frac{1}{2}k_{12}[1]^2 + k_{23}[1][2] + k_{24}[2]^2 + k_{46}[2][4]$$

$$-\frac{d[3]}{dt} = -k_{23}[1][2] + k_{36}[3]^2 - 2k_{63}[6_{na}] + k_{elongation}[6_{i-1}]\frac{2}{i-1}[3]^2$$

$$-\frac{d[4]}{dt} = -\frac{1}{2}k_{24}[2]^2 + k_{46}[2][4] - \frac{3}{2}k_{64}[6_{na}]$$

$$-\frac{d[6_{na}]}{dt} = -\frac{1}{2}k_{36}[3]^2 - k_{46}[2][4] + k_{63}[6_{na}] + k_{64}[6_{na}] + k_{sequestration}[6_{i-1}]\frac{2}{i-1}[6_{na}] + k_{nucleation}[6_{na}]^2$$

$$-\frac{d[6_2]}{dt} = -k_{nucleation}[6_{na}]^2$$

$$-\frac{d[6_i]}{dt} = -ik_{sequestration}[6_{i-1}]\frac{2}{i-1}[6_{na}] + ik_{sequestration}[6_i]\frac{2}{i}[6_{na}] - \frac{i}{2}k_{elongation}[6_{i-1}]\frac{2}{i-1}[3]^2$$
$$+\frac{i}{2}k_{elongation}[6_i]\frac{2}{i}[3]^2 + k_{breakage(i)}[6_i] - k_{breakage(2i)}[6_{2i}]$$

**Supplementary Note 3 | Simulation results for the fibre growth/breakage model vs. removal of breakage**

The plots in Figures 6b and c are obtained from the full plots of the logarithm of replication rates against the logarithm of replicator concentration along full numerical simulations shown in Supplementary Figure 4, in which models involving, or excluding, fibre breakage, respectively, are considered. An autocatalytic "replication" region in the central portion of the latter graphs is surrounded by two regions in which additional effects are noticeable, the first one due to a (zero-order) nucleation rate being significant at lower replicator concentrations ("nucleation" region, with a slope smaller than the one of the main central replication region) and the second one due to the artificially imposed constant food supply not any longer capable of sustaining exponential replication at higher replicator concentrations ("saturation", flat region, where the amount of replicator formed is limited either by the constant food supply at every time step or by the maximum fibre length being reached). The central regions are the ones of interest in order to determine *r* and are the ones reproduced in Figures 6b,c.

Note that the order in replicator was *not* built into our simulations but is a true outcome of these simulations. In experimental systems and also in our simulations there are two processes that impact on the overall observed order in replicator. The first process is the uncatalysed formation of replicator. This process is not autocatalytic and therefore zero order in replicator. The second process is autocatalytic and it is the order in replicator in this process that is relevant for evolutionary scenarios. The overall replication order that is determined experimentally or in our simulations can be regarded as a weighted average of the two

processes. In the limiting case that all “replicator” is produced through the uncatalysed pathway the order in replicator will be zero. In the other limiting case that all replicator is produced through the autocatalytic pathway, the order in replicator may be exactly 1 (or exactly 0.5 in the case of parabolic replicators). In intermediate situations one obtains an order between 0 and 1 (or 0.5). In fact, the observed order in replicator changes during the replication process. If one starts off the replication reaction without any replicator the first molecules of replicator can only be produced through the uncatalysed pathway and the process will be zero-order in replicator at $t = 0$. As more replicator gets produced the autocatalytic pathway will become increasingly efficient, while the uncatalysed pathway will not increase in efficiency. Thus as the reaction progresses the order in replicator will converge on the order of the actual replication reaction. This is exactly the behaviour we observe in our simulations, as shown in Supplementary Figure 4: in what we have termed the nucleation phase the observed order in replicator is smaller than 1 (converging on zero for $t = 0$), while during the more advanced stages of replication (the replication phase) the amount of replicator produced by the uncatalysed pathway becomes increasingly insignificant compared to the amount produced autocatalytically and therefore the order in replicator converges on 1. In between the two phases we observe replication orders that change from 0 to 1. Note that our experimental system is quite unique in that the uncatalysed formation of replicator is very slow. In the absence of fibre breakage (no agitation) we do not observe significant quantities of replicator over a period of 1 month. Thus the uncatalysed pathway contributes very little to the overall replicator production as soon as self-replication has started.

### Supplementary Methods 1 | C++ code

```
//-------------------------------------------------------------------------------------------------------------------------------------------------
// :: KinSim3 ::
//
// Author: Elio Mattia
// Date: February 2011 (v1)
// Required files: none
//
// The second version has a different model with respect to KinSim: the first version bypasses solution hexamers and allows direct nucleation
// of a stack of two hexamers from solution.
// The 2.0 version doesn't allow for nucleation from trimers and tetramers and considers hexamers in solution, which form from trimers and tetramers.
// Only solution hexamers can nucleate, from hexamers, then two growth mechanisms are considered: sequestration of hexamers from solution (which is
// exponential at first, then can become linear, rate-limited by the rate of solution-hexamers formation from the library) and catalyzed synthesis
// of hexamers, just from trimers as a first model, which is exponential if fiber breakage is considered.
//
// VERSION HISTORY
// 2.0 -
// 2.1 - Flag "steady" added: it allows, if on, to maintain a steady state of monomer to its initial concentration, instead of it being consumed
// 2.2 - Added backward reactions from 6 towards 3 and 4
// 3.0 - Allows calculation of P thanks to outputting log(d[R]/dt) against log[R], the slope is P, the order of reaction. "steady" must be on.
// 3.1 - Introduced "steady6" flag, keeps steady the amount of **solution** hexamers. Keeping steady monomer doesn't allow calculation of P
// 3.2 - Introduced "steady3" flag, useful for catalyzed elongation simulations for the calculation of P
// 3.3 - Introduced fiber length distribution histograms calculation and printout
// 3.3a - Not really a version, set maxlen to 100 and thr1 to 10 instead of maxlen/2, longer simulations with different breakage probability than the
standard one
// 3.4 - Introduced 'nucleationon' flag, to (de)activate nucleation from solution hexamers; IMPORTANT: if deactivated, automatically seeds the initial
system with a const distrib of 0.1 to all fiber lengths
// 3.5 - Allowing, thanks to "fiberfeedback", for fiber concentration to influence breakage rate. Law considers a term as independent from fiber
concentration and another one as dependent from it.
//-------------------------------------------------------------------------------------------------------------------------------------------------

#include <math.h>
#include <stdio.h>
#include <stdlib.h>
#include <string.h>
#include <time.h>
```

```
//============================================================ :: Typedef Struct ::
==============================================================
typedef struct configuration {
    float s1;
    float s2;
    float s3;
    float s4;
    float s6;
    float *stacks;
} configuration;


//=========================================================== :: Global Constants ::
=============================================================
const int v1=3;
const int v2=5;
const char vl=' ';
//make these variables when passing to list of duties and cycle the whole main by reading the list
const int maxlen=100;
const int maxlength=maxlen+1;
const long maxtime=10000;
const float monomerinitconc=1;
const float trimerinitconc=2e2;
const float hexamerinitconc=1e1;
const int steady=0;
const int steady3=1;
const int steady6=1;
const float rate12=1e-1;
const float rate23=1e-1;
const float rate24=3e-1;
const float rate36=3e-1;
const float rate46=3e-1;
const float rate63=1e-5;
const float rate64=1e-5;
const float nuclprob=1e-6;
const float sequrate=0;
const float elonrate=3e-1;
const int nucleationon=1;
const int breakageon=0;
const int fiberfeedback=0;
const int fft=10;
const int thr1=maxlen/2;
const int thr2=maxlen;

//============================================================== :: Prototypes ::
================================================================
void simulate(float *,configuration *);
void cfgcopy(configuration *,configuration *);
void generatebp(float *);
configuration *create();
void destroy(configuration *);
void createpath(char *);
void write(long,FILE *,configuration *,float *,float *,float *);
void parse(FILE *,char *);


//=============================================================================================
===================================================
//=============================================================================================
===================================================
//================================================================= :: MAIN ::
===================================================================
//=============================================================================================
===================================================
//=============================================================================================
===================================================
int main() {
   //================================================= :: structures and structured lists ::
=====================================================
   configuration *cfg;
   //========================================================= :: local variables ::
=============================================================
   long t;
   char path[250];
   char txtpath[254];
   FILE *output;
   float bp[maxlength];
   int i;
   float r61,r62,r63;


   //=============================================================== :: code ::
==================================================================

//=============================================================================================
===============================================
   generatebp(bp);
```

```
    createpath(path);
    strcpy(txtpath,path);
    strcat(txtpath,".txt");
    output=fopen(txtpath,"w");
    fprintf(output,"dynamics_%d%c%d N%d T%d C%.0e 12_%.0e 23_%.0e 24_%.0e 36_%.0e 46_%.0e 63_%.0e 64_%.0e NP%.0e SR%.0e ER%.0e
break%d
steady%d\n",v1,'.',v2,maxlen,maxtime,monomerinitconc,rate12,rate23,rate24,rate36,rate46,rate63,rate64,nuclprob,sequrate,elonrate,breakageon,stead
y);

fprintf(output,"dynamics_%d%c%d\tN%d\tT%d\tC%.0e\t12_%.0e\t23_%.0e\t24_%.0e\t36_%.0e\t46_%.0e\t63_%.0e\t64_%.0e\tNP%.0e\tSR%.0e\tE
R%.0e\tbreak%d\tsteady%d\n\n",v1,'.',v2,maxlen,maxtime,monomerinitconc,rate12,rate23,rate24,rate36,rate46,rate63,rate64,nuclprob,sequrate,elonr
ate,breakageon,steady);
    fprintf(output,"%c\t%d\t%d\t%d\t%d\t%d%c%c%c%c\t%d%c%c%c%c\t\t%d%c%c%c%c\t",'t',1,2,3,4,6,' ','s','o','l',6,' ','f','i','b',6,' ','t','o','t');
    for (i=1;i<maxlength;i++) fprintf(output,"\t%d",i);
    fprintf(output,"\t\tlog[R]\tlog(d[R]/dt)\t");
    for (i=1;i<maxlength;i++) fprintf(output,"\t%d",i);
    fprintf(output,"\n\n");

    r61=r62=r63=0;
    cfg=create();
    cfg->s1=monomerinitconc;
    cfg->s3=trimerinitconc;
    cfg->s6=hexamerinitconc;
    t=0;
    write(t,output,cfg,&r61,&r62,&r63);


    for (++t;t<=maxtime;t++) {
        simulate(bp,cfg);

        //Move information relevant to calculation of p
        r61=r62;
        r62=r63;

        //Print results
        write(t,output,cfg,&r61,&r62,&r63);
    }

    destroy(cfg);

    fclose(output);
    output=fopen(txtpath,"r");
    parse(output,path);
    fclose(output);

    return 0;
}

//==========================================================================================
==================================================
//============================================================ :: SIMULATE ::
=================================================================
//==========================================================================================
==================================================
void simulate(float *bp,configuration *cfg) {
    configuration *next;
    int i;
    float totstacks;
    float breakprob;

    next=create();

    //UPDATES: rates proportional to reactants concentrations and kinetic constants
    //update element (increase), then update also the source (decrease),
    //consider stoichiometry: 6mM of 1 equals 3mM of 2, 2mM of 3, 1,5 mM of 4, 1 mM of 6
    //also, 1 mM of 6_2 equals 1mM of fiber ends, 1 mM of 6_3 equals 0,66 mM of fiber ends, 1 mM of 6_4 equals 0,5 mM of fiber ends, etc. (so, it's 2/n)

    //Update 2 (from 1)
    if (cfg->s1>=rate12*(cfg->s1)*(cfg->s1)) {
        next->s2=cfg->s2+rate12*(cfg->s1)*(cfg->s1)/2;
        if (steady) {
            next->s1=cfg->s1;
        } else {
            next->s1=cfg->s1-rate12*(cfg->s1)*(cfg->s1);
        }
    } else {
        next->s2=cfg->s2+cfg->s1/2;
        if (steady) {
            next->s1=cfg->s1;
        } else {
            next->s1=0;
        }
    }
    //Update 3 (from 2 and 1 combined)
    if ((next->s2>=rate23*(cfg->s2)*(cfg->s1))&&(next->s1>=rate23*(cfg->s2)*(cfg->s1))) {
        if (steady3) {
            next->s3=cfg->s3;
        } else {
```

```
        next->s3=cfg->s3+rate23*(cfg->s2)*(cfg->s1);
    }
    next->s2=next->s2-rate23*(cfg->s2)*(cfg->s1);
    if (steady) {
        next->s1=next->s1;
    } else {
        next->s1=next->s1-rate23*(cfg->s2)*(cfg->s1);
    }
} else {
    if (next->s1>=next->s2) {
        if (steady3) {
            next->s3=cfg->s3;
        } else {
            next->s3=cfg->s3+next->s2;
        }
        if (steady) {
            next->s1=next->s1;
        } else {
            next->s1=next->s1-next->s2;
        }
        next->s2=0;
    } else {
        if (steady3) {
            next->s3=cfg->s3;
        } else {
            next->s3=cfg->s3+next->s1;
        }
        next->s2=next->s2-next->s1;
        if (steady) {
            next->s1=next->s1;
        } else {
            next->s1=0;
        }
    }
}
//Update 4 (from 2)
if (next->s2>=rate24*(cfg->s2)*(cfg->s2)) {
    next->s4=cfg->s4+rate24*(cfg->s2)*(cfg->s2)/2;
    next->s2=next->s2-rate24*(cfg->s2)*(cfg->s2);
} else {
    next->s4=cfg->s4+next->s2/2;
    next->s2=0;
}
//Update 6 (from 3, and from 4 and 2 combined)
if (next->s3>=rate36*(cfg->s3)*(cfg->s3)) {
    if (steady6) {
        next->s6=cfg->s6;
    } else {
        next->s6=cfg->s6+rate36*(cfg->s3)*(cfg->s3)/2;
    }
    if (steady3) {
        next->s3=next->s3;
    } else {
        next->s3=next->s3-rate36*(cfg->s3)*(cfg->s3);
    }
} else {
    if (steady6) {
        next->s6=cfg->s6;
    } else {
        next->s6=cfg->s6+next->s3/2;
    }
    if (steady3) {
        next->s3=next->s3;
    } else {
        next->s3=0;
    }
}
if ((next->s4>=rate46*(cfg->s4)*(cfg->s2))&&(next->s2>=rate46*(cfg->s4)*(cfg->s2))) {
    if (steady6) {
        next->s6=next->s6;
    } else {
        next->s6=next->s6+rate46*(cfg->s4)*(cfg->s2);
    }
    next->s4=next->s4-rate46*(cfg->s4)*(cfg->s2);
    next->s2=next->s2-rate46*(cfg->s4)*(cfg->s2);
} else {
    if (next->s2>=next->s4) {
        if (steady6) {
            next->s6=next->s6;
        } else {
            next->s6=next->s6+next->s4;
        }
        next->s2=next->s2-next->s4;
        next->s4=0;
    } else {
        if (steady6) {
            next->s6=next->s6;
        } else {
```

```
                next->s6=next->s6+next->s2;
            }
            next->s4=next->s4-next->s2;
            next->s2=0;
        }
    }
    //Update 3 (from 6)
    if (next->s6>=rate63*(cfg->s6)) {
        if (steady3) {
            next->s3=next->s3;
        } else {
            next->s3=next->s3+rate63*(cfg->s6)*2;
        }
        if (steady6) {
            next->s6=next->s6;
        } else {
            next->s6=next->s6-rate63*(cfg->s6);
        }
    } else {
        if (steady3) {
            next->s3=next->s3;
        } else {
            next->s3=next->s3+next->s6*2;
        }
        if (steady6) {
            next->s6=next->s6;
        } else {
            next->s6=0;
        }
    }
    //Update 4 (from 6)
    if (next->s6>=rate64*(cfg->s6)) {
        next->s4=next->s4+rate64*(cfg->s6)*3/2;
        if (steady6) {
            next->s6=next->s6;
        } else {
            next->s6=next->s6-rate64*(cfg->s6);
        }
    } else {
        next->s4=next->s4+next->s6*3/2;
        if (steady6) {
            next->s6=next->s6;
        } else {
            next->s6=0;
        }
    }

    //Update conc of all the lengths towards +1 growth: for all other lenghts except 2,
    next->stacks[maxlength-1]=cfg->stacks[maxlength-1]; //working always on next avoids update problems
    for (i=maxlength-1; i>=3; i--) {
        //consider ===> SEQUESTRATION <=== with sequestration rate constant, *FIBER END* CONC ([conc of (n-1)], times 2, divided by n-1), conc of
6
        next->stacks[i-1]=cfg->stacks[i-1];
        if ((next->stacks[i-1]>=sequrate*cfg->stacks[i-1]*2/(i-1)*(cfg->s6)*(i-1))&&(next->s3>=sequrate*cfg->stacks[i-1]*2/(i-1)*(cfg->s6))) {
            next->stacks[i]=next->stacks[i]+sequrate*cfg->stacks[i-1]*2/(i-1)*(cfg->s6)*i;
            //hexamer moles become hexamers-on-stack, and take with them (i-1) times moles of hexamers involved in fibers (i-1) hexamers long,
            //to form fibers i hexamers long
            next->stacks[i-1]=next->stacks[i-1]-sequrate*cfg->stacks[i-1]*2/(i-1)*(cfg->s6)*(i-1);
            if (steady6) {
                next->s6=next->s6;
            } else {
                next->s6=next->s6-sequrate*cfg->stacks[i-1]*2/(i-1)*(cfg->s6);
            }
        } else {
            if (next->s6>=next->stacks[i-1]/(i-1)) {
                next->stacks[i]=next->stacks[i]+next->stacks[i-1]*i/(i-1);
                if (steady6) {
                    next->s6=next->s6;
                } else {
                    next->s6=next->s6-next->stacks[i-1]/(i-1);
                }
                next->stacks[i-1]=0;
            } else {
                next->stacks[i]=next->stacks[i]+next->s6*i;
                next->stacks[i-1]=next->stacks[i-1]-next->s6*(i-1);
                if (steady6) {
                    next->s6=next->s6;
                } else {
                    next->s6=0;
                }
            }
        }
        //and ===> CATALYZED ELONGATION <=== with elongation rate constant, fiber end conc (same as above), conc of 3
        if ((next->stacks[i-1]>=elonrate*cfg->stacks[i-1]*2/(i-1)*(cfg->s3)*(cfg->s3)/2*(i-1))&&(next->s3>=elonrate*cfg->stacks[i-1]*2/(i-1)*(cfg-
>s3)*(cfg->s3))) {
            next->stacks[i]=next->stacks[i]+elonrate*cfg->stacks[i-1]*2/(i-1)*(cfg->s3)*(cfg->s3)/2*i;
            //half of trimer moles become hexamers, and take with them (i-1) times moles of hexamers involved in fibers (i-1) hexamers long,
            //to form fibers i hexamers long
```

```
            next->stacks[i-1]=next->stacks[i-1]-elonrate*cfg->stacks[i-1]*2/(i-1)*(cfg->s3)*(cfg->s3)/2*(i-1);
            if (steady3) {
                next->s3=next->s3;
            } else {
                next->s3=next->s3-elonrate*cfg->stacks[i-1]*2/(i-1)*(cfg->s3)*(cfg->s3);
            }
        } else {
            if (next->s3>=next->stacks[i-1]*2/(i-1)) {
                next->stacks[i]=next->stacks[i]+next->stacks[i-1]*i/(i-1);
                if (steady3) {
                    next->s3=next->s3;
                } else {
                    next->s3=next->s3-next->stacks[i-1]*2/(i-1);
                }
                next->stacks[i-1]=0;
            } else {
                next->stacks[i]=next->stacks[i]+next->s3/2*i;
                next->stacks[i-1]=next->stacks[i-1]-next->s3/2*(i-1);
                if (steady3) {
                    next->s3=next->s3;
                } else {
                    next->s3=0;
                }
            }
        }
    }


    //for length 2, consider nucleation rate and conc of 6
    if (nucleationon) {
        if (next->s6>=nuclprob*(cfg->s6)*(cfg->s6)) {
            next->stacks[2]=next->stacks[2]+nuclprob*(cfg->s6)*(cfg->s6);
            if (steady6) {
                next->s6=next->s6;
            } else {
                next->s6=next->s6-nuclprob*(cfg->s6)*(cfg->s6);
            }
        } else {
            next->stacks[2]=next->stacks[2]+next->s6;
            if (steady6) {
                next->s6=next->s6;
            } else {
                next->s6=0;
            }
        }
    }

    //calculate total fiber concentration
    totstacks=0;
    for (i=1; i<maxlength; i++) {
        totstacks+=cfg->stacks[i];
    }

    //Update conc of lower lengths upon breakage of higher lengths: rate proportional to probability (high number approx: we
    //don't consider probability distributions, but just rates), update higher length, then lower length(s)
    if (breakageon) {
        for (i=2; i<maxlength; i++) {
            if (fiberfeedback) {
                breakprob=(bp[i]*(1+totstacks/fft)>1)?1:(bp[i]*(1+totstacks/100000));
            } else {
                breakprob=bp[i];
            }
            if (!(i%2)) {
                next->stacks[i/2]=next->stacks[i/2]+breakprob*next->stacks[i];
                next->stacks[i]=next->stacks[i]-breakprob*next->stacks[i];
            } else {
                next->stacks[(int)(i/2)]=next->stacks[(int)(i/2)]+breakprob*next->stacks[i]*(i-1)/(2*i);
                next->stacks[(int)(i/2)+1]=next->stacks[(int)(i/2)+1]+breakprob*next->stacks[i]*(i+1)/(2*i);
                next->stacks[i]=next->stacks[i]-breakprob*next->stacks[i];
            }
        }
    }


    cfgcopy(cfg,next);
    destroy(next);
}


//=============================================================================================
=================================================
//============================================================= :: CFGCOPY ::
===============================================================
//=============================================================================================
=================================================
void cfgcopy(configuration *c1,configuration *c2) {
    int i;
```

```
    c1->s1=c2->s1;
    c1->s2=c2->s2;
    c1->s3=c2->s3;
    c1->s4=c2->s4;
    c1->s6=c2->s6;

    for (i=0; i<maxlength; i++) {
        c1->stacks[i]=c2->stacks[i];
    }
}

//==========================================================================================
================================================
//================================================================ :: GENERATEBP ::
============================================================
//==========================================================================================
================================================
void generatebp(float *bp) {
    int i;

    //Linear ramp 0-1 from thr1 to thr2, zeroing before
    for (i=1; i<=thr1; i++) {
        bp[i]=0;
    }
    for (; i<=thr2; i++) {
        bp[i]=(float)(i-thr1)/(float)(thr2-thr1);
    }
}

//==========================================================================================
================================================
//================================================================== :: CREATE ::
==============================================================
//==========================================================================================
================================================
configuration *create() {
    configuration *cfg;
    int i;

    cfg=(configuration *)(malloc(sizeof(configuration)));
    cfg->s1=0;
    cfg->s2=0;
    cfg->s3=0;
    cfg->s4=0;
    cfg->s6=0;
    cfg->stacks=(float *)(malloc(maxlength*sizeof(float)));
    for (i=0;i<maxlength;i++) {
        cfg->stacks[i]=nucleationon?0:0.1;
    }

    return cfg;
}

//==========================================================================================
================================================
//================================================================= :: DESTROY ::
==============================================================
//==========================================================================================
================================================
void destroy(configuration *cfg) {
    free(cfg->stacks);
    free(cfg);
}

//==========================================================================================
================================================
//=============================================================== :: CREATEPATH ::
=============================================================
//==========================================================================================
================================================
void createpath(char *txtpath) {
    FILE *lastpath;

    lastpath=fopen("lastpath.txt","w");
    fprintf(lastpath,"dyn%d%c%d%c N%d T%d 1C%.0e 3C%.0e 6C%.0e 12_%.0e 23_%.0e 24_%.0e 36_%.0e 46_%.0e 63_%.0e 64_%.0e NP%.0e
SR%.0e ER%.0e n%d b%d ff%d fft%d 1t%d 2t%d 1s%d 3s%d
6s%d",v1,'.',v2,vl,maxlen,maxtime,monomerinitconc,trimerinitconc,hexamerinitconc,rate12,rate23,rate24,rate36,rate46,rate63,rate64,nuclprob,sequrate
,elonrate,nucleationon,breakageon,fiberfeedback,fft,thr1,thr2,steady,steady3,steady6);
    fclose(lastpath);

    lastpath=fopen("lastpath.txt","r");
    fgets(txtpath,250,lastpath);
    fclose(lastpath);
}

//==========================================================================================
================================================
```

```
//============================================================================ :: WRITE ::
==============================================================================
//==============================================================================================================
============================================================
void write(long t,FILE *output,configuration *cfg,float *r61,float *r62,float *r63) {
    int i;
    float totstacks;
    float tot;

    totstacks=0;
    for (i=1;i<maxlength;i++) {
        totstacks+=cfg->stacks[i];
    }
    tot=cfg->s1+2*cfg->s2+3*cfg->s3+4*cfg->s4+6*cfg->s6+6*totstacks;
    *r63=6*totstacks;
    fprintf(output,"%d\t%f\t%f\t%f\t%f\t%f\t%f\t\t%f\t",t,cfg->s1,2*cfg->s2,3*cfg->s3,4*cfg->s4,6*cfg->s6,6*totstacks,tot);

    for (i=1;i<maxlength;i++) {
        fprintf(output,"\t%f",cfg->stacks[i]);
    }

    if (t>=2) {
        if ((*r63!=*r61)&&(*r62!=0)) {
            fprintf(output,"\t\t%f\t%f\t",(float)(log((double)(*r62))),(float)(log((double)(*r63)-(double)(*r61))));
            for (i=1;i<maxlength;i++) {
                if (totstacks!=0) {
                    fprintf(output,"\t%f",100/totstacks*(cfg->stacks[i]));
                }
            }
        }
    }

    fprintf(output,"\n");
}

//==============================================================================================================
============================================================
//============================================================================ :: PARSE ::
==============================================================================
//==============================================================================================================
============================================================
void parse(FILE *output,char *path) {
    FILE *final;
    char xlspath[154];
    char character;

    strcpy(xlspath,path);
    strcat(xlspath,".xls");
    final=fopen(xlspath,"w");

    while (!(feof(output))) {
        fscanf(output,"%c",&character);
        if (character=='.') {
            fprintf(final,",");
        } else {
            fprintf(final,"%c",character);
        }
    }

    fclose(final);
}

//==============================================================================================================
//==============================================================================================================
//================================================ :: END OF CODE :: ================================================
//==============================================================================================================
//==============================================================================================================
```
